# Anatomic physeal‐sparing MPFL reconstruction in skeletally immature patients shows favourable outcomes at a minimum of 24‐month follow‐up

**DOI:** 10.1002/jeo2.70063

**Published:** 2024-10-22

**Authors:** Panagiotis Ntagiopoulos, Pierrenzo Pozzi, Georgios Kalinterakis, Dimitris Fligkos, Triantafyllia Dimou, Riccardo Compagnoni, Paolo Ferrua, Pietro Simone Randelli

**Affiliations:** ^1^ Hip and Knee Unit Mediterraneo Hospital Athens Greece; ^2^ U.O.C. 1° Clinica Ortopedica, ASST G. Pini‐CTO Milan Italy; ^3^ Università degli Studi di Milano Italy; ^4^ Department of Biomedical, Surgical and Dental Sciences Università degli Studi di Milano Milan Italy; ^5^ Department of Biomedical Sciences for Health Università degli Studi di Milano Milan Italy

**Keywords:** knee, MPFL, paediatric surgery, patella dislocation

## Abstract

**Purpose:**

Recurrent patellar dislocation is a prevalent orthopaedic issue among active paediatric and adolescent populations. Bony surgical procedures are not recommended in growing patients; therefore, the focus of surgery is on restoring the medial patellar ligaments, with different reconstructive techniques available. This retrospective case series focuses on the 2‐year outcomes of medial patellofemoral ligament (MPFL) reconstruction in skeletally immature patients with open physis.

**Methods:**

Twenty‐four consecutive patients with patellofemoral instability and open growth plates underwent anatomic MPFL reconstruction with a physeal‐sparing technique. All subjects have had more than three episodes of true patellar dislocations. Preoperative radiographic examination included anteroposterior and lateral views to assess patella alta and limb alignment. Magnetic resonance imaging was performed to evaluate trochlear dysplasia and tibial tubercle–trochlear groove (TT–TG) distance. The patients were questioned regarding complications and clinical outcomes using the visual analogue scale (VAS), Kujala and Paediatric International Knee Documentation Committee (Pedi‐IKDC) score. Variables were evaluated using paired *t* test with significance at *p* < 0.05.

**Results:**

The mean age at the time of operation was 13.04 years (9–16 years). The cohort was followed for a mean duration of 38.66 months (24–86 months). The mean time from injury to surgery was 50.45 days (16–80 days). No growth arrest, limb‐length discrepancies or angular deformities were observed post‐operatively during the whole follow‐up period. No patellar re‐dislocations were recorded throughout the study period. The VAS score improved significantly from 5.67 (4–8) to 1.88 (0–4) (*p* < 0.01). The Kujala score improved significantly from 64.67 (44–81) preoperatively to 87.58 (77–100) post‐operatively (*p* < 0.01). The Pedi‐IKDC also increased significantly from 58.81 (34.80–77.70) preoperatively to 90.64 (70.70–100) post‐operatively (*p* < 0.01). The vast majority of patients (87.5%) returned to their pre‐injury activity level. Boys scored better than girls in VAS, Pedi‐IKDC and Kujala score post‐operatively, but these differences were not statistically significant.

**Conclusion:**

Physeal‐sparing MPFL reconstruction in children and adolescents yields excellent midterm results and allows patients to return to sports without redislocation of the patella. Boys scored better than girls in VAS, Pedi‐IKDC and Kujala score post‐operatively, but these differences were not statistically significant.

**Level of Evidence:**

IV: case series with no comparative group.

**Abbreviations**
MPFL: medial patellofemoral ligament
MRI: magnetic resonance imaging
Pedi‐IKDC: Paediatric International Knee Documentation Committee
ROM: range of movement
TT‐TG: tibial tubercle–trochlear groove
VAS: visual analogue scale

## INTRODUCTION

Patellar dislocation in skeletally immature paediatric and adolescent patients is a critical orthopaedic concern, characterized by its increased frequency and its potential for recurrence. A recent population‐based study reported an annual incidence rate of lateral patellar dislocations at 2.58 per 100,000 individuals, with a higher occurrence in females and patients under 19 years of age [15]. Notably, two thirds of these injuries were associated with sports activities and up to 50% of these patients were at risk of recurrent dislocations [27]. The condition in adults is known to lead to long‐term complications, such as osteoarthritis and impaired knee function [30].

The pathophysiology of paediatric patellar dislocation often involves a combination of anatomical and functional factors, which include anatomic predispositions like trochlear dysplasia, patella alta, increased tibial‐tubercle to trochlear groove (TT–TG) distance, and generalized ligamentous laxity [9]. These anatomical features contribute significantly to the instability of the patella and are frequently found in patients with recurrent dislocations. The risk for recurrence increases from 14% in first‐time dislocators with none of the above risk factors, to 88% in patients with all four factors [10].

The evaluation of acute patellar instability begins with a physical exam, focusing on patellar tracking, the presence of J‐sign, joint hyperlaxity, and lower limb alignment [11]. Standard imaging includes standing anteroposterior (AP) and lateral knee X‐rays, as well as patellar axial views. The aim is to check for patellar subluxation, possible osteochondral fractures, trochlear dysplasia, and patellar height [19]. Magnetic resonance imaging (MRI) is crucial to determine the medial patellofemoral ligament (MPFL) injury's location and extent, assess osteochondral damage, identify trochlear dysplasia, discover loose bodies, and measure the TT–TG distance [17].

The choice between conservative and surgical treatment depends on a thorough evaluation of the injury and its morphology. Risk factors for recurrence after the first dislocation have been studied extensively. Balcarek et al. proposed a ‘Patellar Instability Severity Score’ assessing factors like age, bilaterality, trochlear dysplasia, patellar tilt and height, and TT–TG distance. A score above 4 out of 7 indicates a high recurrence risk, favouring surgical intervention [1]. Traditionally, conservative treatment was considered the standard of care in first‐time dislocators, especially in younger ages (<9 years old), unless a fracture or an osteochondral avulsion was present [35]. However, the results of conservative management were not consistent, particularly when taking the primary factors of patellar instability into account. A recent meta‐analysis of randomized controlled trials showed lower redislocation rates and higher Kujala scores for patients treated with surgery [25]. Consequently, there has been a well‐documented increase in surgically addressing first‐time dislocators, including those who are skeletally immature [18].

The MPFL, a well‐known patellar stabilizer, is always compromised in cases of patellar dislocation, and its insufficiency significantly contributes to the recurrence of the pathology in this patient population [8]. Current management approaches for acute patellar dislocations are evolving, with a growing emphasis on early surgical intervention to prevent recurrent instability [13]. This includes MPFL reconstruction or repair, which has shown promising outcomes in preventing the recurrence of instability and mitigating the risk of progressive cartilage damage [3]. Given the fact that additional bony procedures should be performed in skeletally immature patients only in the presence of severe rotational abnormalities [6] or valgus deformity [28], in most cases patellar instability can be addressed solely by soft‐tissue procedure, and therefore accuracy and efficacy in an isolated MPFL reconstruction is of vital importance [29]. Several physeal‐sparing techniques for MPFL reconstruction have been described; they obtain patellar stability and prevent potential damage to the growth plates [24]. This article examines the efficacy and safety of dynamic MPFL reconstruction, a technique adapted for young patients with open physis, using a semitendinosus autograft and a mini‐open patella gliding tunnel.

## MATERIALS AND METHODS

This retrospective case series study was conducted on 24 patients (16 girls and 8 boys) with age ranging from 9 to 16 years in the period between September 2016 and September 2023. All surgeries were performed in the same hospital by a senior surgeon after approval by our institution's scientific research board (54/20‐06‐2016). All patients and their parents were informed and consented to their participation in the study.

Pre‐operative imaging included standing AP and lateral radiographs and axial patellar views, long‐leg films for the whole lower limb, and patellar height was measured on true lateral X‐rays using the Caton–Deschamps ratio. MRI scans were obtained for all patients to assess skeletal deformities, limb axis and intra‐articular pathology, to measure TT–TG distance, and to assess trochlear dysplasia. Inclusion criteria for the study were: (1) more than three episodes of true documented patellar dislocation before to surgery and unimproved despite a non‐operative treatment programme or (2) patellar dislocation with loose osteochondral bodies. The exclusion criteria were: (1) patients with closed physis, (2) patients having undergone prior ipsilateral knee surgery, and (3) patients with underlying syndromes causing intellectual or physical disabilities. In more than half of the included patients, the dislocation happened during sport‐related activities. The presence of possible trochlear dysplasia was documented, was explained to the patient and to the parents, and was benignly neglected, since only soft‐tissue procedures can be performed in this population.

In the context of MPFL reconstruction, especially in paediatric and adolescent patients, outcome measurement scales like the visual analogue scale (VAS), Kujala Anterior Knee Pain Scale, and the Paediatric International Knee Documentation Committee (Pedi‐IKDC) have been used for assessing the effectiveness and patient satisfaction post‐surgery. The VAS is a simple yet effective tool for measuring pain intensity. The Kujala Anterior Knee Pain Scale is more specific to patellofemoral disorders. It encompasses various aspects such as pain, stiffness, swelling, and limitations in activities like climbing or squatting [19, 26]. Finally, the Pedi‐IKDC is an adapted version of the International Knee Documentation Committee's scale, tailored for younger patients [23]. This comprehensive scale evaluates knee symptoms, the ability to participate in sports activities, and overall knee function [16]. For paediatric patients undergoing MPFL reconstruction, these scales offer a detailed view of how the surgery affects their knee function and ability to return to normal activities, especially sports. The evaluation method to exclude a possible skeletal growth disturbance was done with clinical assessment.

### Surgical technique

**Figure 1:**
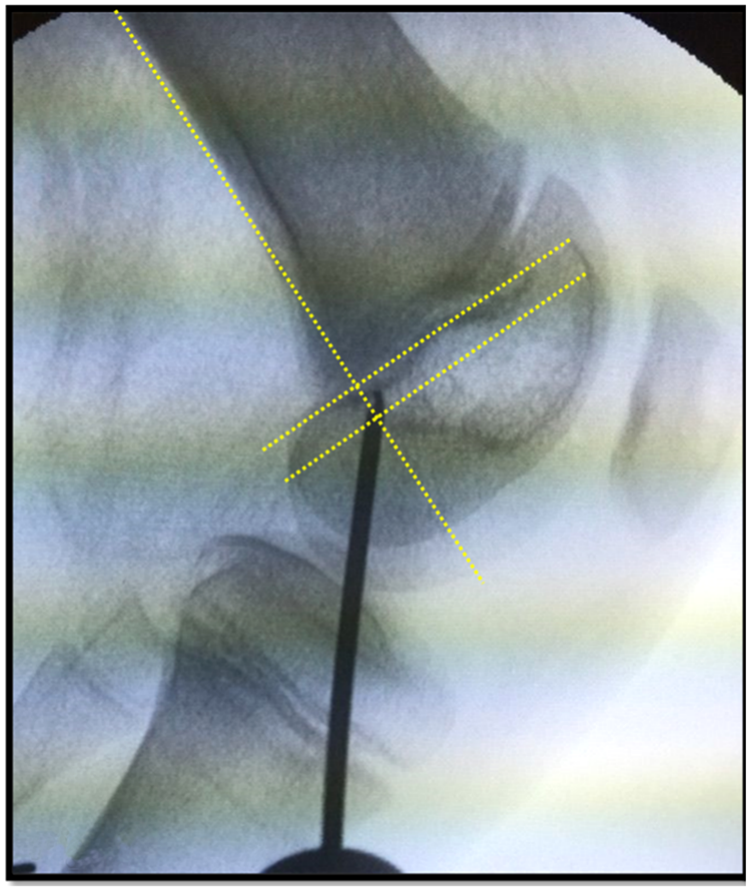
Targeting of Schöttle point under fluoroscopy guidance.

**Figure 2:**
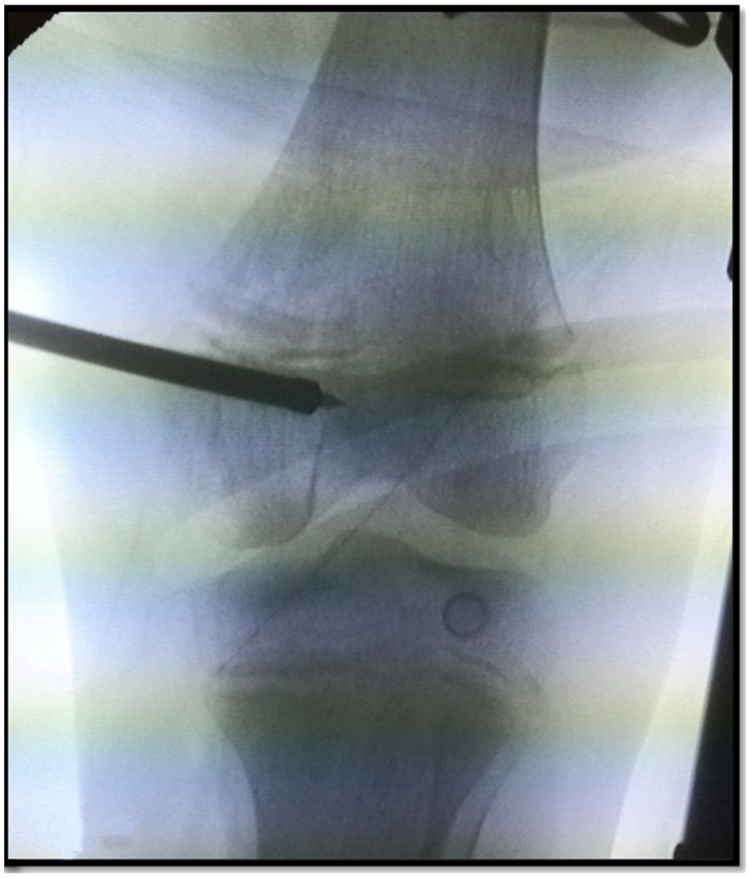
Hand reaming distal to femoral physis.

The surgical technique includes the following steps: Semitendinosus tendon is harvested in a standard fashion, and arthroscopy follows in all cases for removal of loose body, cartilage damage assessment, and lateral retinaculum release. A vertical mini open patella approach on the medial border of the patellar allows for the formation of the gliding tunnel of an approximate diameter of 4 mm. The graft is sliding and not fixed in the patellar tunnel. At the femoral level, to avoid growth plate interference, the Schöttle point is accurately targeted by employing fluoroscopic guidance on the sagittal plane (Figure 1 *)*. After verification of the correct position on the sagittal plane, assessment on the coronal plane with fluoroscopy ensures the avoidance of physis damage. A cannulated 6 mm hand reamer is used instead of a power drill, advancing up to 35 mm distal to the femoral physis in a caudal direction *(*Figure 2). Graft passage is performed in a standard fashion using suture passers from the U‐shaped patella tunnel, under the medial retinaculum pulley, subcutaneously to Layer 2 and out to the femoral insertion. Using a suture passer, the graft is inserted in the femoral blind tunnel, and the attaching sutures are temporarily secured with a Kocher clamp on the lateral surface of the femur. Then, the knee is cycled ten times for pre‐tensioning. Lateral patella translation is assessed in full knee extension and the desired firm endpoint stop of translation and a translation of less than 2 patellar width quadrants is achieved by further graft tensioning or loosening (by adjusting the sutures in the Kocher clamp). When isometric pull‐out forces are obtained, the graft is finally fixed in 70° of flexion with an appropriately sized interference screw of 7 mm.

### Rehabilitation

Early rehabilitation protocols were used in all cases. Post‐operatively, patients are allowed to maintain partial weight‐bearing with knee ROM from 0° to 60° in a hinged brace for the first 2 weeks, progressively increased to 90° until Week 4, and finally unrestricted. From Weeks 6 to 12, isometrics, short arc quads, and electrical stimulation are started, with progression to stationary bike and elliptical. Jogging is initiated after 3 months, with return to sport by 5–6 months.

### Statistical analysis

Descriptive statistics are presented for numerical demographic data, and the categorical variables are reported as percentages. The values of preoperative and postoperative VAS, Kujala and Pedi‐IKDC were compared using the Student's *t* test. All data were analysed using SPSS version 26. A *p* value < 0.05 was considered statistically significant.

## RESULTS

Patients' demographic and radiological characteristics are depicted in Table 1. In total, 24 patients were enroled in the study. The female‐to‐male ratio was 3:1, namely 16 girls and 8 boys. Each patient had experienced more than three episodes of true patellar dislocation before surgery, indicating a chronic and recurrent nature of their condition. The predominant cause of these dislocations was sports‐related injuries, accounting for 54% of the cases. School activities were the second most common cause, responsible for 29% of the injuries, followed by direct trauma, which accounted for the remaining 16%.

**Table 1:** Patients' demographic and radiological characteristics.

Age, mean (SD)	13.04 (2.29)
No. of girls	16
No. of boys	8
Causes of dislocation, No.	
Sports related	13
School activities	7
Direct trauma	4
TT–TG distance, mean (SD), mm	14.15 (1.85)
Caton‐Deschamps index	1.04 (0.14)
Trochlear dysplasia, No.	
Type A	17
Type B	7
Type C	–
Type D	–
Time between the initial trauma and the surgical intervention, mean (SD), days	50.45 (18.18)
Return to play, mean (SD), months	7.3 (1.25)

Abbreviations: M, mean; SD, standard deviation; TT–TG, tibial tuberosity–trochlear groove.

The mean age at the time of the operation was 13.04 (9–16). The mean time between the initial trauma and the surgical intervention was approximately 1.5 months (mean: 50.45 days and SD: 18.18). The mean follow‐up period was 38.66 months with a range from 24 to 86 months. The mean patella height by the Caton–Deschamps index was 1.04 (0.8–1.25) with two children demonstrating patella alta with a Caton–Deschamps of 1.23 and 1.25, respectively. The mean TT–TG was 14.15 (11.23–18.00). Seventeen patients demonstrated mild dysplasia (Dejour Type A) and 7 of them had Dejour B dysplasia. Preoperative physical examination showed a positive apprehension sign in all patients while the J‐sign was positive in seven patients. Out of 24 patients, 25% of them presented with loose bodies in the knee joint, a factor that can exacerbate joint instability and pain. Additionally, preoperative cartilage damage was observed in a sizeable portion of the cohort, with 67% showing damage on the medial patella and 25% on the lateral femoral condyle.

Twenty‐one patients (87.5%) returned at least to their preoperative level of injury whereas 3 patients (2 girls/1 boy) failed to reach the pre‐injury level. Notably, all three patients mentioned that the main reason had been the fear of re‐injury. For those who return to sports, the mean time from the operation to return to play was 7.3 months. There were no patellar re‐dislocations recorded throughout the study period. Six patients had a remaining apprehension sign with no other further symptoms. Regarding skeletal growth, there were no instances of growth arrest, limb‐length discrepancies, or angular deformities recorded. Regarding complications, four patients presented with reduced knee flexion (flexion range: 65–90°) at the end of the 6th week, but they regained full range of movement (flexion >120°) with rigorous physical therapy programme after 3 weeks; one patient developed a superficial wound infection which was treated successfully with oral antibiotics.

In elaborating on the statistical analysis of the study, the focus lies on two key tables: one comparing pre‐ and post‐operative scores and the other examining post‐operative results with sex as the independent variable. Table 2 presents a comprehensive comparison of patients' scores before and after undergoing MPFL reconstruction. The data show statistically significant improvements in post‐operative scores, indicating the surgery's success in enhancing knee stability and functionality.

**Table 2:** Preoperative and post‐operative, VAS, Kujala and Pedi‐IKDC scores.

	Preoperatively	Post‐operatively (final follow‐up)			
Score	Μ (SD)	Μ (SD)	*t* test	*p*	Cohen's *d*
VAS	5.67 (1.09)	1.88 (1.08)	25.76**	1.8226E−18	5.25
Kujala	64.67 (10.98)	88.57 (7.46)	−18.71**	2.05E−15	3.81
Pedi‐IKDC	58.81 (13.9)	90.64 (8.07)	−17.69**	6.8176E−15	3.61

Abbreviations: M, mean; Pedi‐IKDC, Pediatric International Knee Documentation Committee; SD, standard deviation; VAS, visual analogue scale.

** *p* < 0.01.

Table 3 delves into the post‐operative outcomes with a specific focus on gender differences. The analysis reveals a trend where males tend to score higher than females in post‐operative assessments, although the result is not statistically significant.

## DISCUSSION

In the present retrospective study, anatomic reconstruction of the MPFL in patients with open growth plates resulted in a significant improvement in the average Kujala score from 64.67 preoperatively to 87.58 post‐operatively, and in the average Pedi‐IKDC score from 58.81 to 90.64, with 87.5% of patients returning to their pre‐injury activity level, demonstrating satisfying outcomes for this technique. Numerous ways to surgically manage patella instability in skeletally immature patients have been described in the literature, with a special emphasis on MPFL reconstruction; distal and proximal realignment procedures are contraindicated in this population, except for very selected cases since they can lead to growth disturbances or early‐onset arthritis [13, 16, 29]. These procedures fall into two categories: anatomic and non‐anatomic. MPFL reconstruction is considered an anatomic procedure, and the importance of respecting the patellar and femoral insertion of the native ligament during the reconstruction has been noted by several authors [22]. However, in patients with open growth plates, different technical issues need to be considered. Due to the adjacent physis, femoral fixation of the MPFL is challenging. Thus, most of the described techniques in children and adolescents are not strictly anatomic, as they use the femoral origin of the MCL or of the adductor magnus tendon insertion as a reference for the femoral fixation [2, 5]. One study conducted by Lind et al. revealed a concerning revision rate of 21% in the paediatric patient cohort when performing an MPFL reconstruction technique with soft tissue femoral graft fixation [14]. Nelitz et al. were the first who report the use of a strictly anatomic technique, based on anatomical and radiological studies by Kepler et al. [12] and Schοttle et al. [33]. He reported no recurrent dislocations and improved Kujala scores [22]. Our study confirms those excellent results, as no recurrent patella instability was noticed.

As far as patellar fixation is concerned, a double‐bundle MPFL reconstruction with two divergent tunnels on the anteromedial and proximal half of the patella was performed. The graft is sliding and not fixed in the patella tunnel; this can favour the occurrence of micromotion, undesired because of graft slackening [36]. However, in our study, no signs of graft slackening and no further re‐dislocations were noticed. This technique allowed us to recreate the fan shape of MPFL and, at the same time, to respect the anatomical patellar site, as well as decrease the risk of fractures. In fact, no patellar fractures were reported in our study group. The average age at the time of surgery was 13 years, ranging from 9 to 16 years, a value in line with previous studies [4, 34]. Furthermore, the majority of patients included in the study were females; this demographic reflects the higher incidence of patellar instability among female adolescents as described in the literature [31].

The average duration between the initial trauma and the surgical intervention was approximately 1.5 months. This relatively short time frame between injury and treatment indicates a proactive approach to addressing the instability. Given that in this cohort, 67% of the patients reported experiencing instability during routine daily activities, while the remaining 33% experienced instability solely during sports, an early treatment is crucial, so that the patient can return to pre‐injury activity levels.

In terms of preoperative conditions, 25% of the patients presented with loose bodies in the knee joint, a value higher than the 12% reported in a recent meta‐analysis [21]. Additionally, preoperative cartilage damage was observed in a significant portion of the cohort, with 67% showing damage on the medial patella and 25% on the lateral femoral condyle. Multiple studies [7, 24, 37] reported that cartilage damage is frequently observed in patients with both first time and repeated patellar dislocations: they highlighted the severity of the joint damage in patients undergoing MPFL reconstruction. An arthroscopic assessment of the articular surface and an eventual surgical intervention are therefore necessary for restoring knee function and stability.

Over an extensive follow‐up period averaging 24 months, with a range from 9 to 84 months, these patients were closely monitored to assess the outcomes of the MPFL reconstruction. A longer follow‐up period is crucial for evaluating both the intermediate and long‐term efficacy of the surgical intervention. Sappey‐Mariner et al. [32], in a series of 211 MPFL reconstructions, report a 4.7% failure rate with the necessity of reintervention. The mechanism of failure was traumatic for every patient, and it happened at a mean follow‐up time of 3 years. The long follow‐up period is crucial for evaluating both the immediate and long‐term efficacy of the surgical intervention. Among our patients, 87.5% returned to the same level of sports as preoperatively. These findings confirm the results of a recent systematic review showing that 87% of the children and adolescents who performed MPFL reconstruction for recurrent patella instability returned to the same level of play [20].

Table 3 describes post‐operative outcomes with a specific focus on gender differences. Here, the analysis reveals a trend where males tend to score higher than females in post‐operative assessments. This observation might be reflective of differing responses to surgery between genders, potentially influenced by anatomical, physiological, or even lifestyle differences. However, this observed difference in scores between males and females is not statistically significant. This indicates that while there is a visible trend, it does not hold substantial statistical weight to conclusively assert gender‐based differences in outcomes. The lack of statistical significance underscores the consistency of the surgical procedure's effectiveness across genders, further validating its application in a diverse paediatric and adolescent population.

**Table 3:** Post‐operative VAS, Kujala and Pedi‐IKDC scores according to sex.

	Female	Male			
Post‐operatively scores	M (SD)	M (SD)	*t* test	*p*	Cohen's *d*
VAS	1.94 (0.99)	1.75 (1.16)	0.41	0.29	0.17
Kujala	86.88 (7.56)	89 (7.54)	−0.65	0.91	0.28
Pedi‐IKDC	89.84 (8.77)	92.25 (6.68)	−0.68	0.47	0.29

*Note*: *p* < 0.05.

Abbreviations: M, mean; Pedi‐IKDC, Pediatric International Knee Documentation Committee; SD, standard deviation; VAS, visual analogue scale.

In summary, this technique allows for a successful and safe MPFL reconstruction, in the absence of severe postoperative complications. No growth arrest, limb‐length discrepancies, or angular deformities were reported in the follow‐up period. These statistical insights show the efficacy of the surgical technique but also highlight its applicability across different genders, marking it as a versatile approach in treating patellar instability in young patients.

A major advantage of this study is that all operations were performed by a single surgeon (P.G.N.), as well as the fact that only skeletally immature patients with confirmed open physis were included. Additionally, all patients were assessed preoperatively with plain radiographs and MRI, evaluating possible risk factors for patellofemoral instability. Another strength point is the long follow‐up, as re‐dislocations typically occur within 3 years after surgery, as mentioned above. That being said, there are some limitations to be mentioned. First, its retrospective design makes finding a comparison group extremely difficult. Second, the sample size was relatively small. Moreover, while all patients followed the same rehabilitation protocol, rehabilitation was conducted at different locations. Finally, these results reflect the outcomes of patients at a single hospital and, as such, might not be generalizable to the broader orthopaedic community.

## CONCLUSION

The described anatomic physeal‐sparing MPFL reconstruction technique presents a promising surgical option for paediatric and adolescent patients with open physis and recurrent patellar instability. This study's findings, alongside existing literature, support the safety and efficacy of this approach, particularly in eliminating the risk of growth plate damage. Nevertheless, continued research, ideally through longitudinal studies with larger cohorts, is essential to fully understand the long‐term outcomes and optimize treatment strategies for this vulnerable population.

## AUTHOR CONTRIBUTIONS

*Conception of the study*: Panagiotis Ntagiopoulos, Paolo Ferrua, Pierrenzo Pozzi and Georgios Kalinterakis. *Acquisition of data*: Panagiotis Ntagiopoulos and Georgios Kalinterakis. *Analysis of data*: Panagiotis Ntagiopoulos and Georgios Kalinterakis. *Drafting of the work*: Pierrenzo Pozzi, Paolo Ferrua, Panagiotis Ntagiopoulos and Georgios Kalinterakis. *Revising it clinically for important intellectual content*: Panagiotis Ntagiopoulos, Paolo Ferrua, Riccardo Compagnoni, Pietro Simone Randelli, Triantafyllia Dimou and Dimitris Fligkos. *Final approval of the version to be published*: Pierrenzo Pozzi, Georgios Kalinterakis, Panagiotis Ntagiopoulos, Paolo Ferrua, Riccardo Compagnoni, Pietro Simone Randelli, Triantafyllia Dimou and Dimitris Fligkos.

## CONFLICT OF INTEREST STATEMENT

The authors declare no conflict of interest.

## ETHICS STATEMENT

This study was approved by the ethical committee of Mediterraneo Hospital, Athens, Greece. All patients and their parents were informed and consented to their participation in the study.

## Data Availability

Data outside the one reported in the manuscript are available upon request from the authors.
